# Supplementary material for: Deep Learning Cerebellar Magnetic Resonance Imaging Segmentation in Late-Onset GM2 Gangliosidosis: Implications for Phenotype
Source: medRxiv. 2025 Apr 11:2025.04.08.25325262. Preprint. [Version 1] doi: 10.1101/2025.04.08.25325262 (PMC12036421; doi:10.1101/2025.04.08.25325262)
Supplement: Supplement 1 [file NIHPP2025.04.08.25325262v1-supplement-1.pdf]

medRxiv preprint doi: https://doi.org/10.1101/2025.04.08.25325262; this version posted April 11, 2025. The copyright holder for this preprint (which was not certified by peer review) is the author/funder, who has granted medRxiv a license to display the preprint in perpetuity. This article is a US Government work. It is not subject to copyright under 17 USC 105 and is also made available for use under a CC0 license.

Deep Learning Cerebellar Magnetic Resonance Imaging Segmentation in Late-Onset GM2 Gangliosidosis: Implications for Phenotype

Supplementary Material

Table of Contents

Methods……………………………………………………………………………………….…27

Supplement A: Natural History Study Participant Characteristics……………..………...………27

Supplement B: Neurotypical Controls Participants and T1-Weighted Acquisition……...………28

Supplement C: Cerebellar Lobule Segmentation and Cortical Thickness ……………………….30

Supplement D: Cerebellar Lobule Individual Segmentation ……………………..…………...32

Results……………………………………………………………………………………...34

Supplement E. Cerebellar Lobule Volumetric Analysis …….……………………………..…….34

Supplement F. Cerebellar Lobule Cortical Thickness Analysis ………………………………….35

Supplement G: Cerebellar Lobule Volumetric Analysis Estimates and Standard Errors ……..…36

Supplement H: Cerebellar Lobule Cortical Thickness Analysis Estimates and Standard Errors …37

medRxiv preprint doi: https://doi.org/10.1101/2025.04.08.25325262; this version posted April 11, 2025. The copyright holder for this preprint (which was not certified by peer review) is the author/funder, who has granted medRxiv a license to display the preprint in perpetuity. This article is a US Government work. It is not subject to copyright under 17 USC 105 and is also made available for use under a CC0 license.

Supplementary Methods

Supplement A: Natural History Study Participant Characteristics

Table A1. Natural History Study T1-Weighted MRI Scan Age (n = 25). N/A are designated when a participant did not have the above referenced MRI scan. Specific ages were redacted per Medrxiv requirements.

Participant	NHS	Sex	GM2 Sub-type	Baseline Age (years old)	Scan #2 Age (years old)	Scan #3 Age (years old)	Scan #4 Age (years old)	Average T1-weighted interval (years)	Number of T1 Scans
1	32	F	LOTS	26-30	26-30	31-35	N/A	2	3
2	46	F	LOTS	41-45	41-45	41-45	41-45	1	4
3	47	M	LOTS	51-55	51-55	51-55	56-60	1.5	4
4	50	F	LOTS	26-30	26-30	N/A	N/A	2	2
5	103	F	LOTS	41-45	41-45	N/A	N/A	2	2
6	56	F	LOTS	26-30	26-30	26-30	31-35	1.67	4
7	70	M	LOTS	36-40	41-45	41-45	41-45	1.33	4
8	65	M	LOTS	31-35	36-40	N/A	N/A	2	2
9	57	F	LOTS	51-55	56-60	56-60	61-65	2.5	4
10	67	M	LOTS	26-30	31-35	N/A	N/A	2	2
11	55	F	LOTS	26-30	26-30	26-30	31-35	1.67	4
12	37	F	LOTS	31-35	31-35	36-40	N/A	2	3
13	66	M	LOTS	16-20	21-25	N/A	N/A	2	2
14	104	F	LOTS	66-70	N/A	N/A	N/A	N/A	1
15	98	F	LOTS	21-25	N/A	N/A	N/A	N/A	1
16	92	M	LOTS	31-35	N/A	N/A	N/A	N/A	1
17	83	F	LOTS	31-35	N/A	N/A	N/A	N/A	1
18	76	M	LOTS	46-50	N/A	N/A	N/A	N/A	1
19	61	M	LOTS	21-25	N/A	N/A	N/A	N/A	1
20	60	M	LOTS	21-25	N/A	N/A	N/A	N/A	1
21	75	M	LOSD	51-55	56-60	56-60	N/A	2.5	3
22	68	F	LOSD	41-45	41-45	41-45	46-50	1.67	4
23	43	F	LOSD	56-60	61-65	61-65	N/A	1.5	3
24	45	M	LOSD	46-50	51-55	51-55	51-55	1.67	4
25	74	M	LOSD	56-60	56-60	N/A	N/A	2	2

medRxiv preprint doi: https://doi.org/10.1101/2025.04.08.25325262; this version posted April 11, 2025. The copyright holder for this preprint (which was not certified by peer review) is the author/funder, who has granted medRxiv a license to display the preprint in perpetuity. This article is a US Government work. It is not subject to copyright under 17 USC 105 and is also made available for use under a CC0 license.

Supplement B: Neurotypical Control Participant Characteristics

NIMH[2,3]

Participants from "The National Institute of Mental Health (NIMH) Intramural Healthy Volunteer Dataset"[2,3] were scanned using a General Electric (GE) discovery MR750W 3T system with a 32-channel head coil. There were 62 participants with the FSPGR sequence and 92 participants with the MPRAGE sequence.[2,3] With the following parameters:

MPRAGE
TR/TE = 6.95/2.92 ms
Flip angle = 8°
Voxel = 1 mm isotropic voxels

FSPGR
TR/TE = 7.35/3.04 ms
Flip angle = 11°
Slice Thickness = 1.2 mm

Neurocognitive Aging[4,5]

Participants from the "Neurocognitive aging data release with behavioral, structural, and multi-echo functional MRI measures" were included in this analysis.[4,5] MRI data was acquired from one of two sites either the Cornell Magnetic Resonance Imaging Facility in Ithaca, New York or the York University Neuroimaging Center in Toronto, Canada[5]. MRI data from the Cornell University site was acquired on a 3T GE discovery MR750W 3T system with a 32-channel head coil. T1 weighted imaging was acquired with the following parameters: TR/TE = 2530/3.4 ms; 7° flip angle; 1 mm isotropic voxels, and 176 slices. MRI data from York University site was acquired on a 3T Siemens TimTrio MRI scanner with a 32-channel head coil. T1 weighted imaging was acquired from this site with the following parameters: TR/TE = 1900/2.52 ms, flip angle = 9°, 1 mm isotropic voxels, 192 slices.

Paingen_placebo[6,7]

Participants from the "Paingen_Placebo" data set were included in this analysis. MRI data was acquired on a Siemans Prisma 3T system with a 32-channel head coil at the University of Colorado at Boulder.[6] T1 weighted imaging was acquired with the following parameters TR/TE = 2000/2.11 ms, flip angle = 8°, FOV = 256 mm, and resolution = 0.8 × 0.8 × 0.8 mm.

AgeRisk[8,9]

MRI data from the "AgeRisk" data set was acquired using a Siemens 3T MAGNETOM Prisma magnetic resonance imaging (MRI) system and a 20-channel head coil at the University Hospital Basel, Switzerland. T1-weighted MRI data was acquired using a magnetization-prepared rapid gradient echo sequence with the following parameters: TR/TE= 2500/4.25 ms, inversion time = 1100 ms, flip angle = 7°, field of view = 256 mm × 256 mm, 192 slices, voxel dimensions = 1.0 mm isotropic.

medRxiv preprint doi: https://doi.org/10.1101/2025.04.08.25325262; this version posted April 11, 2025. The copyright holder for this preprint (which was not certified by peer review) is the author/funder, who has granted medRxiv a license to display the preprint in perpetuity.
This article is a US Government work. It is not subject to copyright under 17 USC 105 and is also made available for use under a CC0 license.

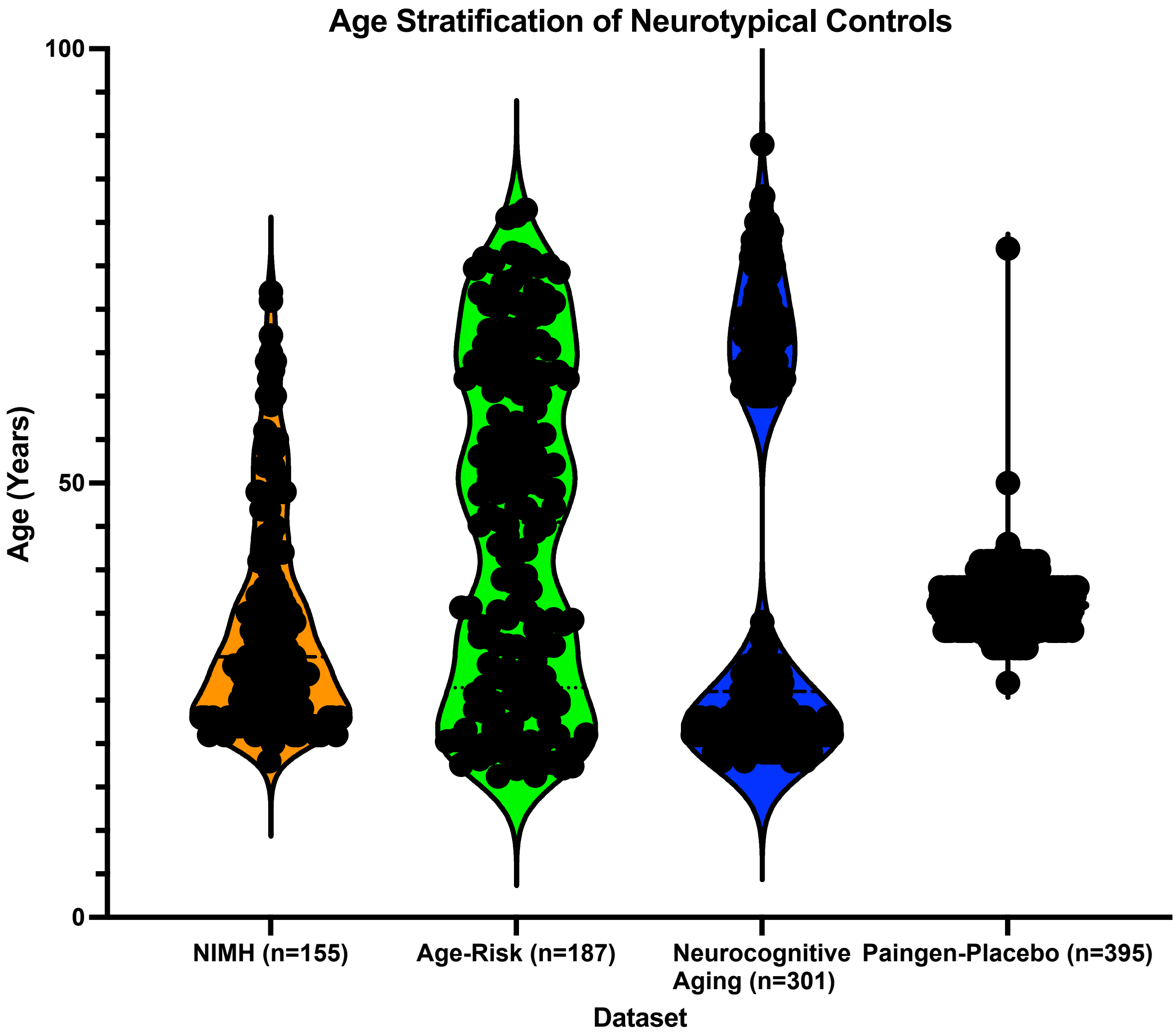

Figure B1. Violin Plot of Neurotypical Controls Age.

medRxiv preprint doi: https://doi.org/10.1101/2025.04.08.25325262; this version posted April 11, 2025. The copyright holder for this preprint (which was not certified by peer review) is the author/funder, who has granted medRxiv a license to display the preprint in perpetuity. This article is a US Government work. It is not subject to copyright under 17 USC 105 and is also made available for use under a CC0 license.

Supplement C: Cerebellar Lobule Segmentation and Cortical Thickness

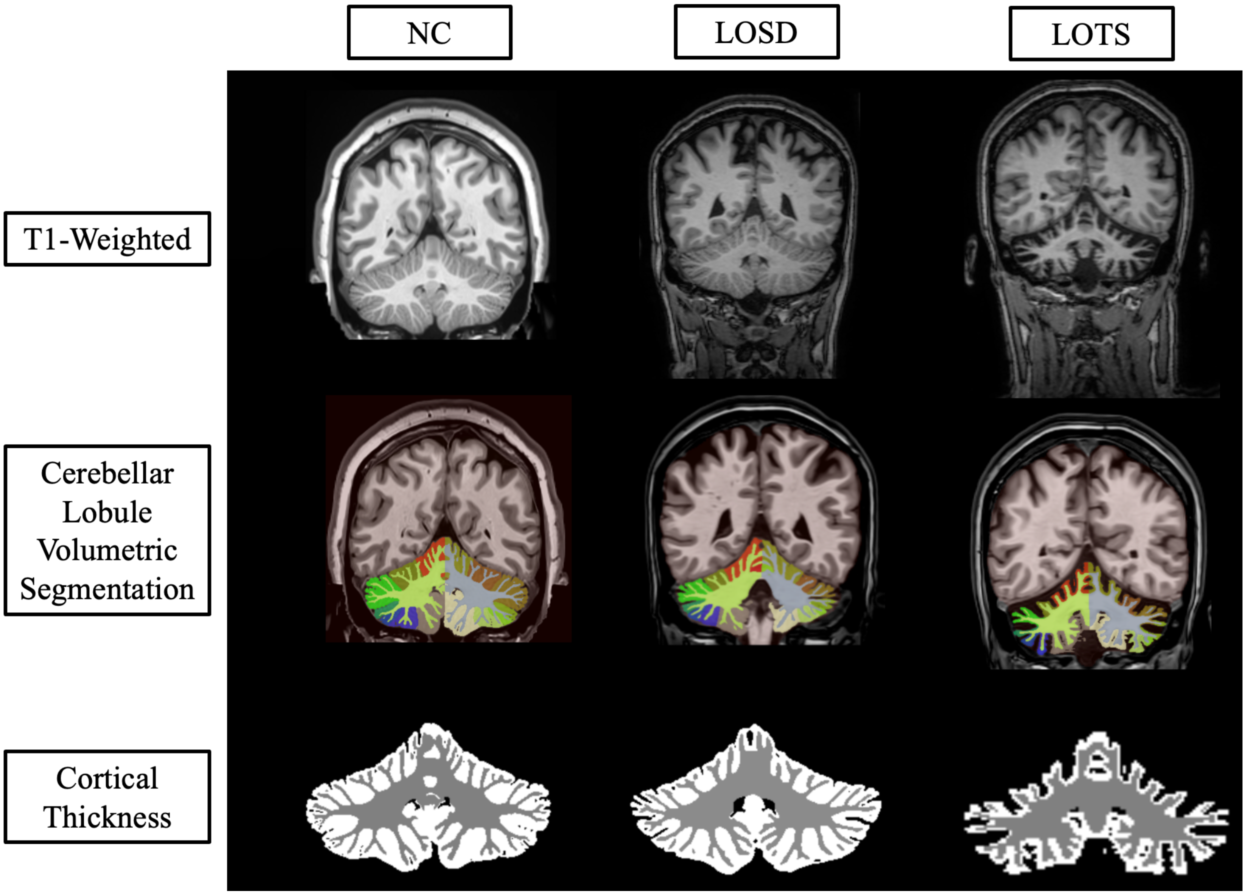

Figure C1. Coronal deep learning segmentation of cerebellar lobules. Column one represents imaging from one 41- to 45-year-old neurotypical control (NC) female from the NIMH volunteer dataset. Column two represents imaging from one 41- to 45-year-old late-onset Sandhoff disease (LOSD) female patient. Column three represents imaging from one 41- to 45-year-old -year-old late-onset Tay-Sachs (LOTS) disease female patient. Row one represents unprocessed T1-weighted imaging for the three participants. Row two represents cerebellar lobule volumetric segmentation at the same slice for the three participants where distinct lobules are separated by color. Row three represents the cortical thickness estimation from *DeepCeres* at the same slice for the three participants where white matter is shown in grey and gray matter is shown in white. Specific ages were redacted per Medrxiv requirements.

medRxiv preprint doi: https://doi.org/10.1101/2025.04.08.25325262; this version posted April 11, 2025. The copyright holder for this preprint (which was not certified by peer review) is the author/funder, who has granted medRxiv a license to display the preprint in perpetuity.
This article is a US Government work. It is not subject to copyright under 17 USC 105 and is also made available for use under a CC0 license.

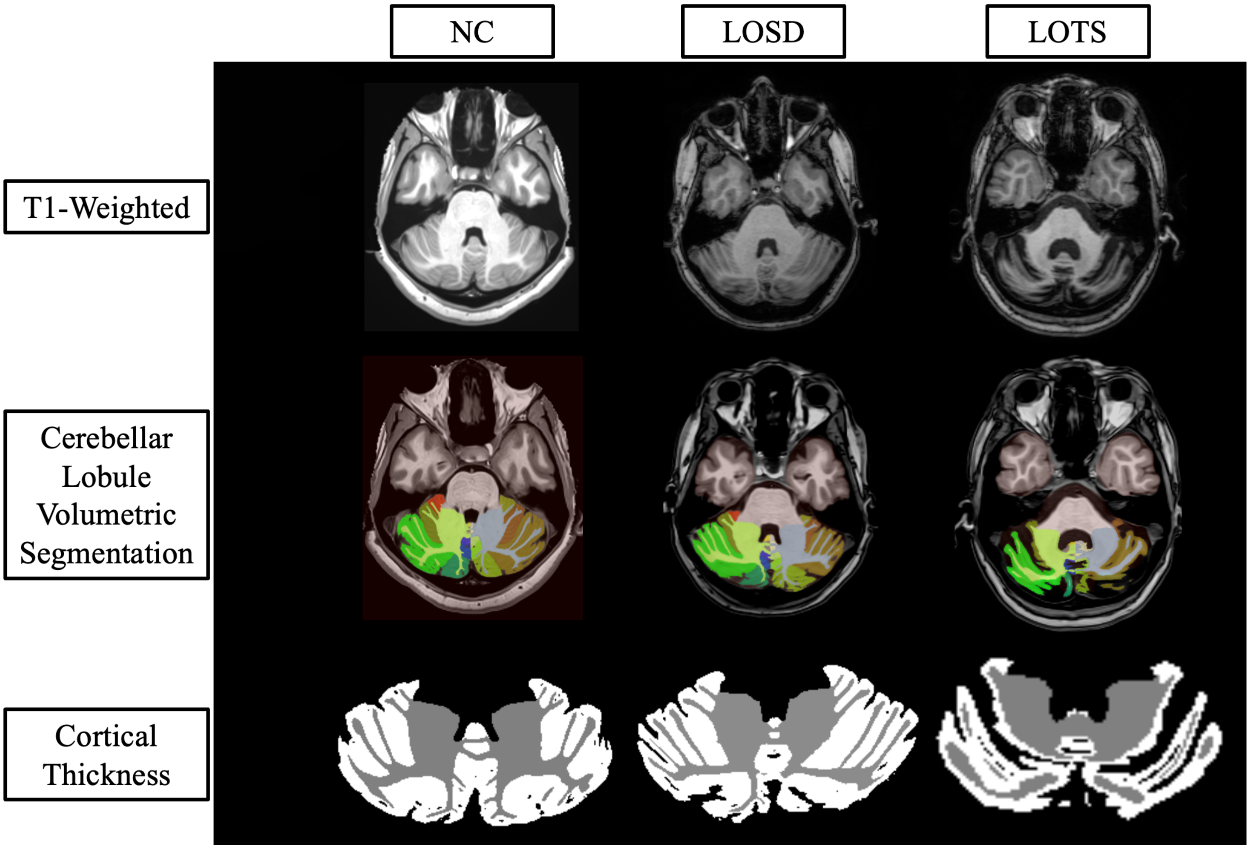

Figure C2. Axial deep learning segmentation of cerebellar lobules. Column one represents imaging from one 41- to 45-year-old neurotypical control (NC) female from the NIMH volunteer dataset. Column two represents imaging from one 41- to 45-year-old late-onset Sandhoff disease (LOSD) female patient. Column three represents imaging from one 41- to 45-year-old late-onset Tay-Sachs (LOTS) disease female patient. Row one represents unprocessed T1-weighted imaging for the three participants. Row two represents cerebellar lobule volumetric segmentation at the same slice for the three participants where distinct lobules are separated by color. Row three represents the cortical thickness estimation from *DeepCeres* at the same slice for the three participants where white matter is shown in grey and gray matter is shown in white. Specific ages were redacted per Medrxiv requirements.

medRxiv preprint doi: https://doi.org/10.1101/2025.04.08.25325262; this version posted April 11, 2025. The copyright holder for this preprint (which was not certified by peer review) is the author/funder, who has granted medRxiv a license to display the preprint in perpetuity.
This article is a US Government work. It is not subject to copyright under 17 USC 105 and is also made available for use under a CC0 license.

Supplement D: Cerebellar Lobule Individual Segmentation

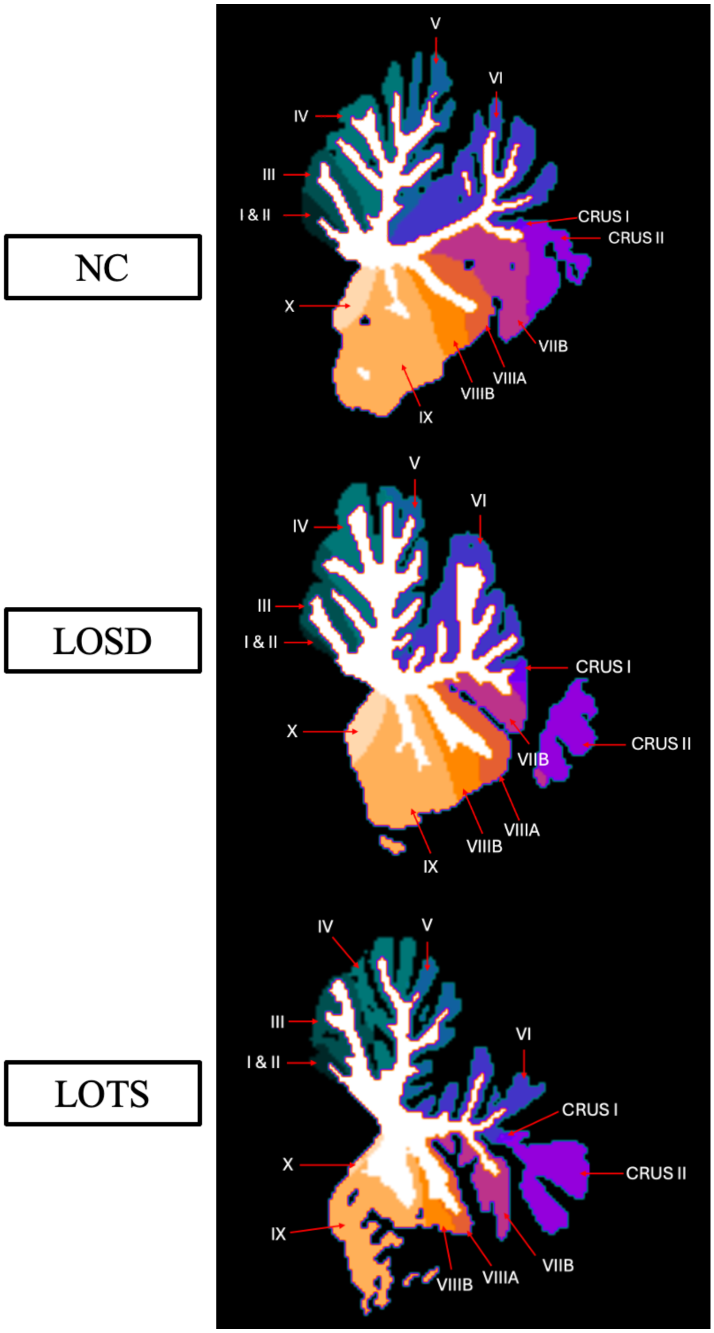

Figure D1. Labeled cerebellar lobule deep learning segmentation. Row one represents imaging from one 41- to 45-year-old neurotypical control (NC) female from the NIMH volunteer dataset. Row two represents imaging from one 41- to 45-year-old late-onset Sandhoff disease (LOSD) female patient. Row three represents imaging from one 41- to 45-year-old late-onset Tay-Sachs (LOTS) disease female patient. Each participants' MRI scan was registered to the MNI space, and a sagittal slice at coordinate x = -2.5 was shown for each participant. Specific ages were redacted per Medrxiv requirements.

medRxiv preprint doi: https://doi.org/10.1101/2025.04.08.25325262; this version posted April 11, 2025. The copyright holder for this preprint
(which was not certified by peer review) is the author/funder, who has granted medRxiv a license to display the preprint in perpetuity.
This article is a US Government work. It is not subject to copyright under 17 USC 105 and is also made available
for use under a CC0 license.

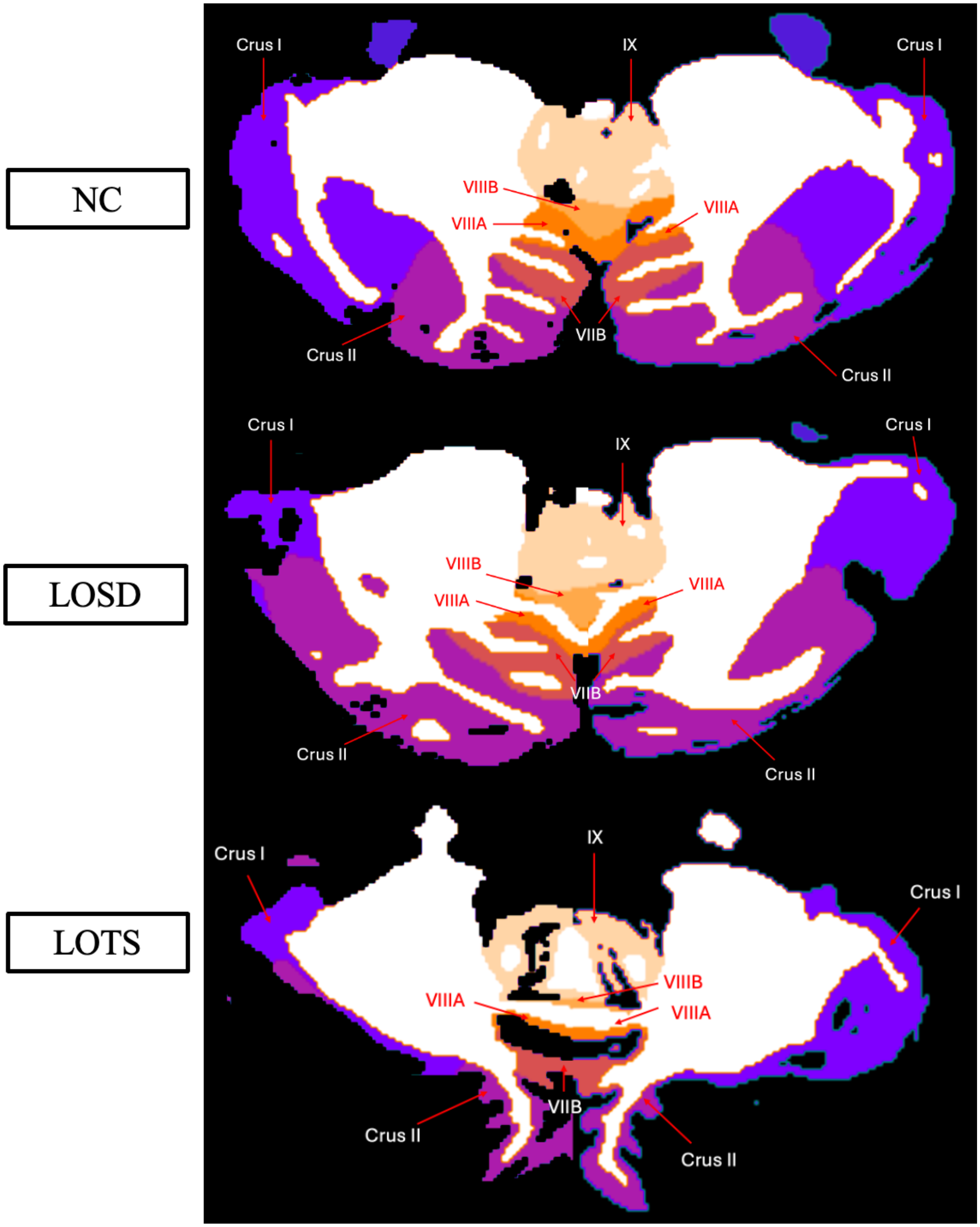

Figure D2. Labeled cerebellar lobule deep learning segmentation. Row one represents imaging from one 41- to 45-year-old neurotypical control (NC) female from the NIMH volunteer dataset. Row two represents imaging from one 41- to 45-year-old late-onset Sandhoff disease (LOSD) female patient. Row three represents imaging from one 41- to 45-year-old late-onset Tay-Sachs (LOTS) disease female patient. Each participants' MRI scan was registered to the MNI space, and an axial slice at coordinate z = -40.5 was shown for each participant. Specific ages were redacted per Medrxiv requirements

medRxiv preprint doi: https://doi.org/10.1101/2025.04.08.25325262; this version posted April 11, 2025. The copyright holder for this preprint (which was not certified by peer review) is the author/funder, who has granted medRxiv a license to display the preprint in perpetuity.
This article is a US Government work. It is not subject to copyright under 17 USC 105 and is also made available for use under a CC0 license.

Supplement E: Cerebellar Lobule Volumetric Analysis

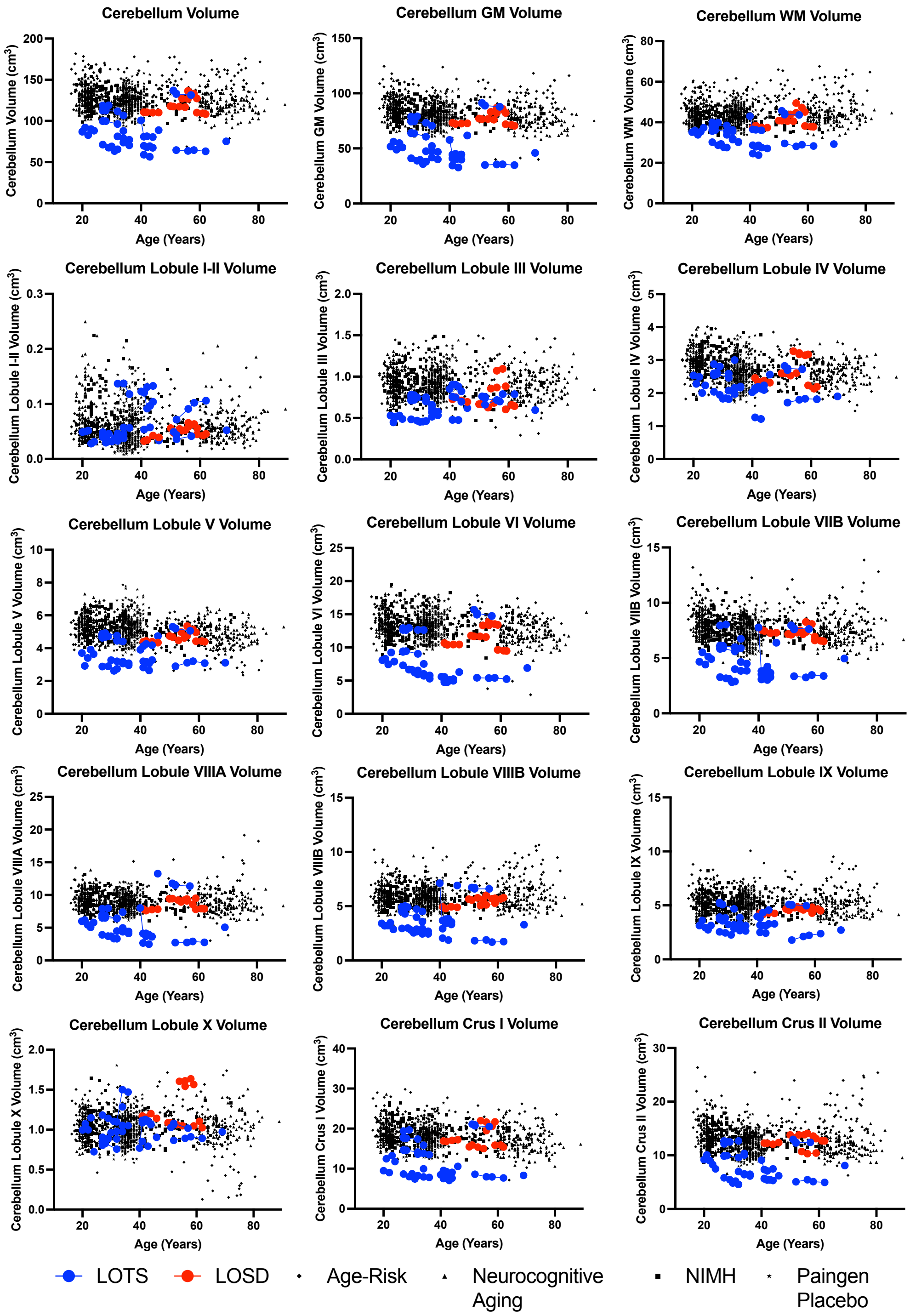

Figure E1. Volumetric Analysis of cerebellar lobule segmentation. Neurotypical controls are shown as individual black points with different symbol shapes for each Open-Source data set. Late-onset Sandhoff Disease (LOSD) patients are shown as red circles with red connecting lines. Late-onset Tay-Sachs (LOTS) disease patients are shown as blue circles with blue connecting lines.

medRxiv preprint doi: https://doi.org/10.1101/2025.04.08.25325262; this version posted April 11, 2025. The copyright holder for this preprint (which was not certified by peer review) is the author/funder, who has granted medRxiv a license to display the preprint in perpetuity.
This article is a US Government work. It is not subject to copyright under 17 USC 105 and is also made available for use under a CC0 license.

Supplement F: Cerebellar Lobule Cortical Thickness Analysis

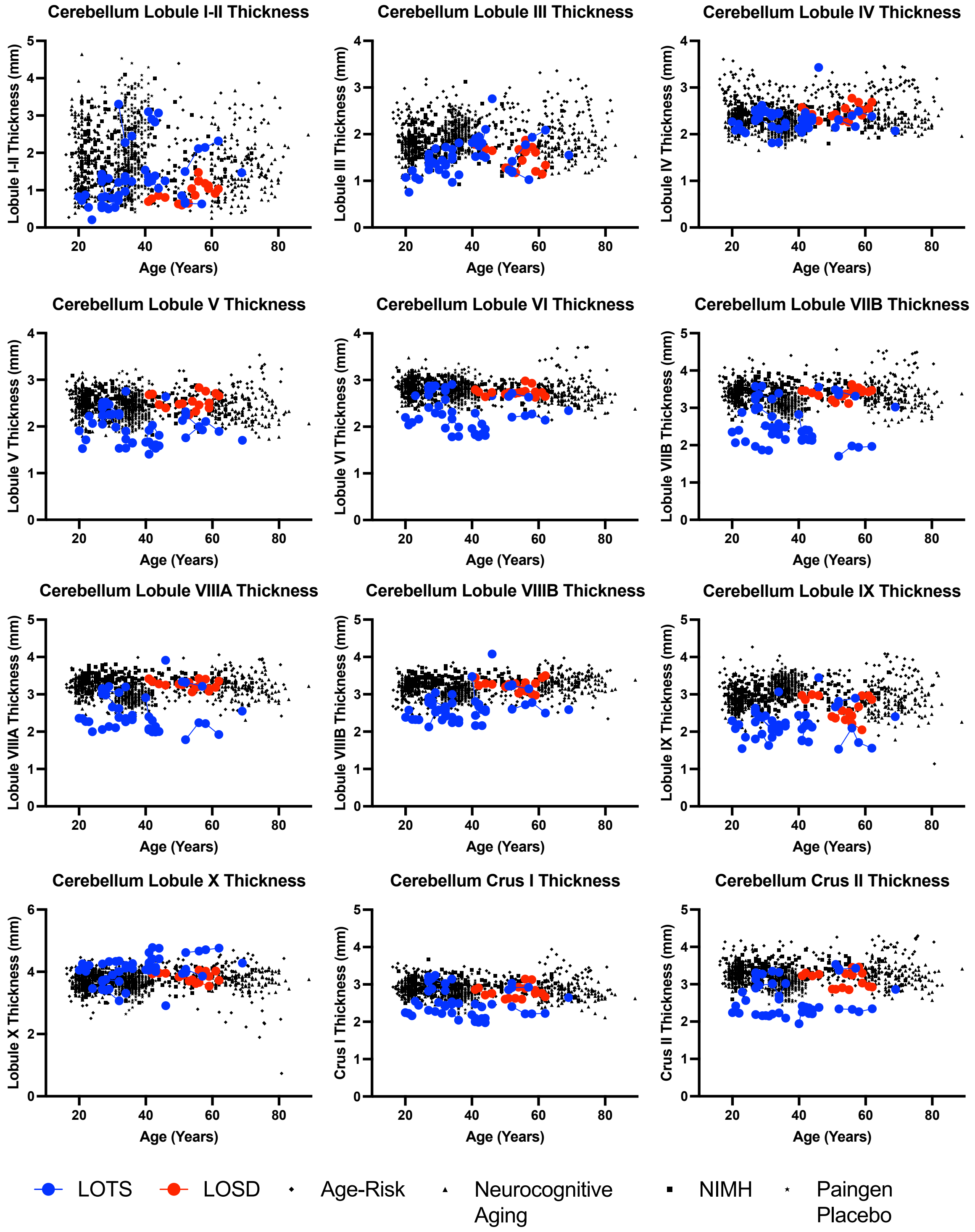

Figure F1. Cortical thickness analysis of the 12 cerebellar lobules. Neurotypical controls are shown as individual black points with different symbol shapes for each Open-Source data set. Late-onset Sandhoff Disease (LOSD) patients are shown as red circles with red connecting lines. Late-onset Tay-Sachs (LOTS) disease patients are shown as blue circles with blue connecting lines.

medRxiv preprint doi: https://doi.org/10.1101/2025.04.08.25325262; this version posted April 11, 2025. The copyright holder for this preprint (which was not certified by peer review) is the author/funder, who has granted medRxiv a license to display the preprint in perpetuity. This article is a US Government work. It is not subject to copyright under 17 USC 105 and is also made available for use under a CC0 license.

Supplement G: Cerebellar Lobule Volumetric Analysis Estimates and Standard Errors.

Table G1. Cerebellar Volumetric MRI Analysis. All volumes were controlled for ICV. Statistical analysis was performed using a linear mixed effects model where *P*-values < 0.01 were considered significant and bolded.

Structure	*LOGG v NC*	*LOSD v NC*	*LOTS v NC*	*LOSD v LOTS*
**Estimate (Standard Error)**				
**Cerebellum**	**-1.966 (0.180)**	-0.245 (0.396)	**-2.402 (0.199)**	**-2.710 (0.712)**
**Cerebellar Gray Matter**	**-1.450 (0.130)**	-0.077 (0.281)	**-1.797 (0.143)**	**-2.019 (0.559)**
**Cerebellar White Matter**	**-0.516 (0.064)**	-0.175 (0.143)	**-0.604 (0.071)**	**-0.665 (0.172)**
**Lobule I-II**	-0.0003351 (0.0005128)	-0.0008 (0.0012)	-0.0002 0.0006	0.002 (0.001)
**Lobule III**	**-0.01638 (0.002458)**	-0.0077 (0.0055)	**-0.019 (0.003)**	-0.007 (0.004)
**Lobule IV**	**-0.01766 (0.005333)**	0.0082 (0.0118)	**-0.024 (0.006)**	-0.030 (0.015)
**Lobule V**	**-0.07231 (0.009154)**	-0.009 (0.020)	**-0.088 (0.010)**	-0.068 (0.029)
**Lobule VI**	**-0.2849 (0.02681)**	-0.069 (0.058)	**-0.339 (0.030)**	-0.308 (0.105)
**Lobule VIIB**	**-0.1309 0.01544**	-0.006 (0.035)	**-0.165 (0.017)**	-0.176 (0.060)
**Lobule VIIIA**	**-0.1574 (0.0178)**	-0.006 (0.039)	**-0.198 (0.020)**	-0.208 (0.104)
**Lobule VIIIB**	**-0.1164 0.01277**	-0.032 (0.030)	**-0.139 (0.014)**	-0.118 (0.055)
**Lobule IX**	**-0.1065 (0.01354)**	-0.037 (0.030)	**-0.124 (0.015)**	**-0.088 (0.029)**
**Lobule X**	0.006788 0.002476	**0.020 (0.006)**	0.003 (0.003)	-0.015 (0.008)
**CRUS I**	**-0.3224 0.04065**	0.039 (0.088)	**-0.414 (0.045)**	**-0.523 (0.142)**
**CRUS II**	**-0.2337 0.03517**	0.019 (0.078)	**-0.297 (0.039)**	**-0.374 (0.095)**

medRxiv preprint doi: https://doi.org/10.1101/2025.04.08.25325262; this version posted April 11, 2025. The copyright holder for this preprint (which was not certified by peer review) is the author/funder, who has granted medRxiv a license to display the preprint in perpetuity.
This article is a US Government work. It is not subject to copyright under 17 USC 105 and is also made available for use under a CC0 license.

Supplement H: Cerebellar Lobule Cortical Thickness Analysis Estimates and Standard Errors.

Table H1. Cerebellar Cortical Thickness MRI Analysis. All volumes were all normalized in relation to the cube root of ICV (adimensional). Statistical analysis was performed using a linear mixed effects model where *P*-values < 0.01 were considered significant and bolded.

Structure	*LOGG v NC*	*LOSD v NC*	*LOTS v NC*	*LOSD v LOTS*
**Estimate (Standard Error)**				
**Lobule I-II**	**-5.666 (1.390)**	-6.828 (3.122)	**-5.356 (1.550)**	4.180 (2.733)
**Lobule III**	**-3.760 (0.6189)**	-3.495 (1.378)	**-3.818 (0.690)**	1.168 (1.740)
**Lobule IV**	-0.04275 (0.5343)	0.898 (1.181)	-0.281 (0.598)	-1.197 (1.333)
**Lobule V**	**-2.66 (0.44)**	0.986 (0.934)	**-3.612 (0.490)**	**-5.389 (1.620)**
**Lobule VI**	**-2.890 0.3439**	-0.017 0.746	**-3.626 (0.380)**	**-4.046 (1.477)**
**Lobule VIIB**	**-4.010 0.537**	0.986 (1.173)	**-5.299 (0.591)**	**-6.922 (2.417)**
**Lobule VIIIA**	**-3.510 0.4656**	1.119 (1.021)	**-4.740 (0.508)**	**-6.346 (2.291)**
**Lobule VIIIB**	**-3.061 0.3739**	0.288 (0.833)	**-4.001 (0.399)**	-4.128 (1.780)
**Lobule IX**	**-6.118 0.5883**	**-3.527 (1.330)**	**-6.806 (0.648)**	-2.501 (1.964)
**Lobule X**	**1.997 (0.5346)**	1.046 (1.179)	**2.258 (0.597)**	1.415 (1.965)
**CRUS I**	**-2.260 (0.397)**	-0.081 (0.869)	**-2.817 (0.441)**	**-4.301 (1.561)**
**CRUS II**	**-3.823 (0.4941)**	-0.317 (1.097)	**-4.715 (0.546)**	-4.405 (1.835)
